# Monthly Digest

**Published:** 1889-05

**Authors:** 


### Monthly Digest.

FOR MAY.

***April Archives, Cosmos, Ohio Journal, Items of Interest, and Headlight; also March Western Journal, International, Review, and Southern Dental Journal, Received too Late for Notice in April Issue.***

The *Southern Dental Journal* contains an excellent and timely editorial on the value to the profession and to individuals of properly conducted dental association meetings and the means of insuring their success. This, the writer says, lies largely in the choice of efficient working officers, and in getting the right men on the different committees. In the choice of officers the thought should be *not* " Whom shall we honor with the place," but " Who will do honor to the place."

The president should be a man of dignified and commanding presence, whose rulings shall be rapid and just; who can make the timid feel at home, and who can and will " squelch the gas-bag." The secretary should stir the members up constantly with reminders, and should " keep the wires hot." Good clinics are all-important; papers should be practical, telling briefly what you did and why you did it.

DENTAL EDUCATION.

Dr. C. B. Hewitt, in his address to the graduating class of the Kansas City Dental College (*Western Journal*), alluding to the separation of the dental from the medical college, said that an expereience of eight years had proved the inadvisablity of taking up the time of the dental student with the general lectures addressed to mixed classes. There are few versatile men; men must be educated specially for their life-work; the dentist must study those things that will assist him to treat diseases that come under his specialty—dental chemistry, dental pathology, dental therapeutics, etc. As a specialist he must have a special education.

Dr. M. C. Marshall, in his address as President of the Arkansas State Dental Association (*Southern Dental Journal*) touches upon the same aspect of the question. He says : " A man with a perfect medical education is no more a dentist than is an educated dentist a physician. There are certain principles that each must understand to practice either profession intelligently, but this does not mean that a man must have a complete medical education to practice dentistry successfully. He adds—I hope to see the day when matriculation will be based upon certain academical qualifications, and graduation upon absolute fitness, be the time spent at college ten weeks or ten years."

SCIENTIFIC AND THEORETIC.—THE GERM THEORY.

The *International* publishes a paper read by Dr. George S. Allan at the Tenth Anniversary Meeting of the Pennsylvania Odontological Society on the Etiology of Caries, in connection with the exhibit of photo micrographs by Drs. Andrews and Sudduth. Admitting all the usual so-called predisposing causes and promoting conditions which *invite* decay but do not *produce* it, Dr. Allan directs attention to two theories, the " Germ Theory," of Dr. Miller, and what he terms the " Protoplasmic Theory " of Drs. Abbott and Heitzman. The latter he pronounces fallacious from the foundation up. The former he accepts as thoroughly accounting for all the conditions found in the mouth. It is grounded on demonstration from beginning to end, every possible source of error having been eliminated by the most careful and painstaking experiments. It accounts for nearly every phase of caries, shows whence comes the acid that dissolves the lime salts of the teeth, explains how the animal basis substance is afterwards destroyed, and how *per force* cavities or pockets are formed in the teeth. The presence of an acid being admitted as an initiatory step, Dr. Miller shows whence it comes, names it, and points out the organism at work manufacturing it, viz : lactic acid, one of the waste products of bacterial life in the presence of a fermentable substance.

In contact with the lime salts of the tooth chemical action takes place, the lactic acid supplants the phosphoric and carbonic acids of the tooth and soluble salts are formed. The organisms would be smothered in their own excreta but for this constant absorption by the limesalts of the tooth. The cavity being thus started, the bacteria follow after, penetrating and enlarging the dentinal tubuli. The production of artificial caries in a sound tooth, by means of a pure culture of the bacteria found in decaying teeth, with identically the same distension of tubuli, breaking down of matrix, formation of caverns, with the same advance—by one of semi-decalcified dentine always found in a carious tooth, furnishes a complete demonstration of the bacterial origin of caries. The germ theory alone can repeat, out of the mouth, the processes that go on within the mouth, and produce an artificial caries simulating perfectly the natural caries of the teeth, fully explaining both the distended tubules and the broken down basis substance.

In an editorial on this subject the *International* cites the rule laid down by Koch, that to establish a claim to the discovery of the germ that produces a certain disease it must be proven *first*, that the germ is the constant accompaniment of the diseased condition; *second*, the discoverer must isolate, inoculate, and reproduce the disease; *third*, he must determine the character of the toxic element that produces the pathological condition.

These precise and exacting conditions have all been complied with by Dr. Miller in determining the etiological connection of a certain microbe with the disease known as dental caries, yet, notwithstanding the positive evidence given, there are many who still refuse to accept the proof of ocular demonstration.

In a paper read before the New York Odontological Society by Robert Ormiston, M.D., (reported in *Cosmos*) he spoke of the epoch produced in the history of medicine by the germ theory which has revolutionized therapeutics and transformed surgery into a new art. Dr. Ormiston emphasized the importance to *the dentists* of putting into every day practice *what they know* in this line, the mouth being a perfect forest of bacterial algea, many of them pathogenic.

Reviewing briefly the various theories heretofore advanced to account for dental caries Dr. Ormiston spoke of the work of Dr.

Miller, of whom he said that he had triumphantly submitted to the discipline of the rules laid down by Koch for the verification of parasitic pathogenic organisms, demonstrating scientifically the parasitic theory of dental caries.

In the review department of the *Western Journal* we find an article from the *Microscope* in which the author—admitting as *demonstrated* the etiological relation of micro-organisms to certain of the infectious diseases, and as *probable* that future observations will establish the specific microbe of each—asks the question, "How do micro-organisms induce disease?" Dismissing as unsatisfactory various theories that have been offered, such as their disorganization of the blood, their production of mycotic thrombi, their consumption of the proteids of the body, their destruction of the blood corpuscles, etc., he says that the production of the ptomaines under the influence of bacterial life, in connection with the ultimate etiology of disease, offers a new field of research which promises wonderful results.

VASCULARITY OF TOOTH SUBSTANCE.

In his analysis of the "protoplasmic" theory of dental caries (*International*) Dr. Geo. S. Allan disputes Dr. Abbott's view that caries is an inflammatory process on the ground that both enamel and dentine are non-vascular, and not influenced by the circulation; consequently no true inflammatory conditions can exist.

In the discussion of Dr. Allan's paper Dr. C. N. Peirce took exception to this basis of argument, claiming that very simple experiments will prove both the existence of organic matter and a circulatory system—the former by the decalcification of an extracted tooth, leaving only the flexible organic basis substance, preserving the contour of the tooth minus its density; the latter may be shown by comparing the weight of a freshly extracted tooth with the same when thoroughly dried, and again, after immersion in a liquid; thus proving its permeability. Changes in the color of the teeth during life; their varying sensibility under different systemic conditions; their varying density at different ages, and other changes, both physiological and pathological, all prove the existence of vascular tissues which hold the teeth in close connection with the arterial and nervous systems.

On this point Dr. R. R. Andrews said—There is certainly a certain amount of circulation through the tooth; there can be no doubt about it; it is not the same circulation as blood circulation, but there is a fluid, and a certain circulation.

Dr. Dwinelle cited in proof of this circulation the injection of dentine, after a severe accident with red coloring matter, presumably from broken down red corpuscles.

PHOSPHITE STARVATION.

The *Archives* publishes a very forcible article from the pen of S. R. Percy, M.D., on the causes of the diseased condition of the teeth so common during pregnancy and indicates very clearly the remedy. The cause being the drain upon the system by the demands of the growing fœtus, the remedy is proper diet and the use of the hypophosphites, which, in cases cited was soon followed by the appearance of phosphates in the urine, of which it had been utterly devoid before treatment.

Dr. Percy condemns very severely the prescription of "Wine of cocoa" which, he says, is not a tonic, but merely a sedative, but one, the use of which is running the most dangerous risks. He very justly adds that when a tonic is needed it should be prepared by an educated pharmacist—not by a wholesale grocer.

DIET AND MASTICATION.

The *Southern Dental Journal* publishes an excellent article on the importance of diet, especially to the growing child. What to eat, how to eat, how often to eat, how much to eat, are questions to which the young mother gives too little attention. Giving solid food to infants, restricting growing children to the regular three, or even two meals of adults, gauging the quantity and quality by that satisfactory to a delicate woman, hurrying children off to school with a hurriedly-bolted unchewed breakfast with a bit of pastry to be as hastily swallowed while worried over unlearned lessons; these are but a few of the evils deplored in this admirable paper which ought to be read and laid to heart by every mother in the land.

### CARE OF THE DECIDUOUS TEETH.

The *Dental Headlight* publishes a brief but valuable paper by Dr. A. S. Willis, on the importance of keeping the temporary teeth in as perfect a state of preservation as possible, both from the effect they exert locally upon the structures in which they are located and in which the permanent teeth are being developed, diseases of the temporary teeth necessarily invading their surroundings, the perfect development of the permanent teeth being impossible amidst abscesses and suppurating sinuses. Upon the perfect condition of the temporary teeth depend also that perfect digestion and assimilation of food so important during the formative and developmental period of childhood and growth. The same care and treatment should be given to the temporary as to the permanent teeth, except that in case of death of the nerve and abscess the root canal of the deciduous tooth should not be filled ; the tooth should be kept disarticulated and isolated by grinding off with corundum wheels from time to time as the tooth is exfoliated, thus supplementing nature in her work of absorption of the roots.

The *Items of Interest* publishes an article on the same subject by Dr. E. J. Church, who emphasizes the importance of impressing upon parents the necessity of thorough cleanliness, and the frequent examination of children's teeth by the dentist. When fillings are necessary they should be made as quickly and with as little pain inflicted as possible, using alloys or cements, replacing the latter as often as necessary.

The *Archives* publishes a paper by C. R. Lightner, M.D., entitled

### EYE WORRY,

Or troubles of vision due to errors of refraction, muscular asthenopia, and other causes giving rise to reflex neurosis such as dizziness, headache, functional derangement of the stomach, heart palpitation, etc. Dr. Lightner emphasizes the importance of having the eyes properly tested in order to secure accurate adjustment of glasses for the correction of the sources of these evils so common among dentists, of whom he very justly says—dental practice demands of its devotee good sight and a good conception of depth; in no other profession is there the same amount of forced eye-work under excessive nerve strain and conditions which allow no time to give the "accommodation" rest.

NEURECTONOMY.

The *Ohio Journal* publishes a paper, read by J. H. Glass, M. D., before the New York State Medical Society, giving the details of an operation by which, for the relief of persistent neuralgia, a section of the inferior branch of the fifth nerve was removed without making any external opening, thus avoiding the almost inevitable cicatricial impairment of facial contour. Using only cocaine as a local anæsthetic, the jaws were widely separated by the ordinary dental prop, an opening was made and the soft parts separated down to and about the nerve, which lies in front of the artery. A Theobald's strabismus hook was passed under the nerve which was seized with Walker's clamp strabismus hook, holding it firmly while a hook bistoury was passed under and the upper section made by a to-and-fro motion. The trunk of the nerve was then drawn up as much as possible and the lower section made—completing the neurectomy. The operation seems to have been ample as there has been no recurrence of the neuralgic symptoms.

Dr. E. C. Kirk and Dr. Dwinelle (*Cosmos*), report several very interesting cases of persistent

NEURALGIA

And in one case, a supposed "cancer," due to impacted cuspids. In one case the tooth was found half an inch back of the anterior alveolar plate; the other two were lying obliquely across the palatine arch.

SENSITIVE DENTINE.

Dr. J. R. Callahan, in a paper read before the Mississippi Valley Dental Association, (*Archives*) after discussing and rejecting various methods of treating sensitive dentine and "nervous patients," commends the administration of nitrous oxide gas, giving but from three to six inhalations sufficient to produce a quieting effect without anæsthesia. Either pain is not felt, or the patient becomes calm and indifferent.

In the discussion of this subject Drs. C. M. Wright, H. A. Smith, A. O. Rawls, M. H. Fletcher, and others spoke of the value of the exercise of the hypnotic power—the influence of mind over mind.

Of the "vibratory theory" Dr. Rawls thought the effect was to prevent reflex action, thus preventing pain; Dr. Fletcher thought the action rather an interference with circulation; Dr. Gray uses hypodermic injections of morphine, but without informing the patient what is used, thereby avoiding all danger of the formation of any "habit."

Dr. Lettig (*Cosmos*) obtunds sensitive dentine with cocaine, making a saturated solution in glycerine; Dr. E. Parmly Brown also uses cocaine—a one per cent. solution in combination with a weak solution of carbolic acid and oil of cloves (*Cosmos*).

BLEACHING TEETH.

Dr. E. C. Kirk, in the *Cosmos*, gives the chemical reaction and *modus operandi* of various agents in use for this purpose—hydrogen peroxide, potassium permanganate, chlorine, aluminum chloride, sulphurous acid, etc.

In the use of permanganate of potash the caution is given not to use too concentrated a solution, or the final treatment with oxalic acid may fail owing to the precipitated manganese dioxide, leaving the tooth worse than at first.

In the use of chlorine care should be taken to remove all gold fillings, (filling the upper third of the root canal with gutta-percha) or the resulting auric chloride will give a permanent purple stain. Distilled water should also be used, or a ferri chloride may be formed. It should also be borne in mind that no metallic instrument should come in contact with a tooth after the use of chlorine. Oxychloride can be manipulated with instruments of bone, hard rubber, or wood. In the use of sulphurous acid 100 gr. sodium sulphite and 70 gr. boracic acid are dessicated separately and then intimately ground together in a warm dry mortar and kept in a tightly stoppered bottle. The cavity being prepared and thoroughly dried is filled with the powder which is then moistened with a drop of warm water and the orifice of the cavity closed with warm gutta-percha. With this method there is no objection to the use of steel instruments. Dr. Kirk cited the case of a very badly discolored central incisor bleached by the latter method, which retained its restored color so completely that after six years he failed to recognize it as a devitalized tooth.

